# The Control of Calcium Metabolism in Zebrafish (*Danio rerio*)

**DOI:** 10.3390/ijms17111783

**Published:** 2016-10-26

**Authors:** Chia-Hao Lin, Pung-Pung Hwang

**Affiliations:** 1 National Institute for Basic Biology, National Institutes of Natural Sciences, Okazaki, Aichi 444-8787, Japan; teleost.tw@gmail.com; 2 Institute of Cellular and Organismic Biology, Academia Sinica, Taipei 11529, Taiwan

**Keywords:** calcium, ionocyte, hormone, zebrafish

## Abstract

Zebrafish is an emerging model for the research of body fluid ionic homeostasis. In this review, we focus on current progress on the regulation of Ca^2+^ uptake in the context of Ca^2+^ sensing and hormonal regulation in zebrafish. Na^+^-K^+^-ATPase-rich cells (NaRCs), the specialized ionocytes in the embryonic skin and adult gills, play a dominant role in Ca^2+^ uptake in zebrafish. Transepithelial Ca^2+^ transport in NaRC, through apical epithelial Ca^2+^ channels (ECaC), basolateral plasma membrane Ca^2+^-ATPase (PMCA), and Na^+^/Ca^2+^ exchanger (NCX), is analogous to mammalian renal and intestinal Ca^2+^-absorption cells. Several hormones were demonstrated to differentially regulate Ca^2+^ uptake through modulating the expression of Ca^2+^ transporters and/or the proliferation/differentiation of NaRC in zebrafish. In addition, the counterbalance among these hormones is associated with the maintenance of body fluid Ca^2+^ homeostasis. Calcium-sensing receptor (CaSR) is expressed in several hormone-secreting tissues in zebrafish, and activated CaSR differentially controls calciotropic hormones. The major principles of Ca^2+^ transport and the hormonal control appear to be conserved from zebrafish to other vertebrates including mammals. The new knowledge gained from zebrafish studies provides new insights into the related issues in vertebrates.

## 1. Introduction

Ca^2+^ is required for diverse intracellular and extracellular physiological activities, such as neurotransmission, muscle contraction, and bone remodeling [1,2]; and therefore vertebrates have to maintain Ca^2+^ homeostasis in body fluids. In terrestrial vertebrates, kidneys and intestines are the main organs for Ca^2+^ uptake. In fishes, the aquatic vertebrates, adult gills and the yolk skin of embryonic stages are the major organs to absorb Ca^2+^ from the aquatic environment [3,4,5]. Ca^2+^ absorption in these organs occurs majorly through two pathways in specific epithelial cells: (1) Paracellular transport, which is the passive diffusion of Ca^2+^ through tight junctions; and (2) Transepithelial transport, which actively transports Ca^2+^ through Ca^2+^ transporters [3,4,5]. The genes of tight junctions are very divergent in fish and their function in ionregulation has not been elucidated completely [6,7]. In contrast, previous studies had clarified the molecular mechanism of transepithelial Ca^2+^ uptake in specific epithelial cells, called ionocytes, in fish [8,9,10,11,12,13].

Zebrafish (*Danio rerio*), a freshwater (FW) teleost, natively lives in south Asia and is a popular laboratory animal. Zebrafish is widely used as an animal model to study the development, genetics, and diseases of humans [14]. Furthermore, zebrafish has been an emerging animal model to explore the regulatory mechanism of ionic homeostasis in body fluid in recent years [15,16,17]. This is because zebrafish has several advantages compared with other species: Firstly, zebrafish can produce several hundred fertilized eggs daily, and the development and life cycle of zebrafish is fast and short. Secondly, the gene expression and function are highly conserved between zebrafish and mammals. Thirdly, zebrafish has a complete genetic database and is applicable to the manipulation of forward and reverse genetics. Finally, approaches of molecular and cellular biology and physiology have been well developed in zebrafish.

Hormonal control is a vital mechanism for body fluid ion regulation. Several hormones (i.e., parathyroid hormone (PTH) and vitamin D) have been well known in the maintenance of Ca^2+^ homeostasis in mammals [1]. However, the function of other hormones (i.e., hydrogen sulfide (H_2_S), calcitonin (CT), isotocin, insulin-like growth factor (IGF), and stanniocalcin-1 (STC-1)) on Ca^2+^ regulation was controversial or unclear. In zebrafish, the function of these calciotropic hormones has been explored in recent years [18,19,20,21,22,23,24]. The expression and/or secretion of calciotropic hormones are responsive to extracellular Ca^2+^ levels. Calcium-sensing receptor (CaSR) in specific endocrine organs senses extracellular Ca^2+^ levels and modulates the gene expression and/or secretion of calciotropic hormones in mammals [25]. The effect of CaSR on hormones in zebrafish has also been defined in recent studies [21,26]. As such, zebrafish studies contribute more physiological background information about the hormonal control of body fluid Ca^2+^ homeostasis, thereby providing a more competent model to explore the related topics in biomedical research. Hence, the present review focuses on an overview of the mechanisms of Ca^2+^ regulation and recent progress of their hormonal control in zebrafish; additionally, we also discuss these findings and some unresolved issues by offering comparisons with studies on other species.

## 2. Ca^2+^ Regulation in Zebrafish

### 2.1. Ca^2+^ Uptake in Zebrafish

**Figure 1:**
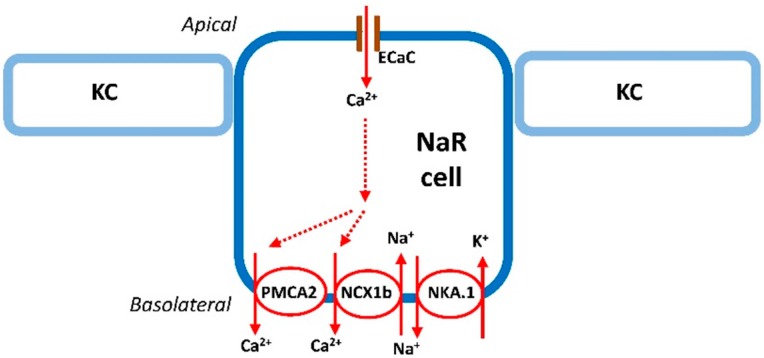
Na^+^-K^+^-ATPase-rich cell (NaRC) in zebrafish. Details of the transport pathways refer to the text (“The mechanism of Ca^2+^ uptake in zebrafish” section). ECaC, epithelial Ca^2+^ channel; KC, keratinocyte; NCX1b, Na^+^/Ca^2+^ exchanger 1b; NAK.1, Na^+^-K^+^-ATPase α1 subunit subtypes (atp1a1a.1); NaRC, Na^+^, K^+^-ATPase-rich cell; PMCA2, plasma membrane Ca^2+^-ATPase 2. Solid and dashed arrows, the route of Ca^2+^ transport in NaRC.

The kidneys and intestine are vital organs for Ca^2+^ absorption in mammals. In specific epithelial cells of these tissues, active transepithelial Ca^2+^ transport is carried out through the operation of apical epithelial transient receptor potential cation channel subfamily 5 and/or 6 (TRPV5 and/or 6), the Ca^2+^ channel, basolateral plasma membrane Ca^2+^-ATPase (PMCA), and Na^+^/Ca^2+^ exchanger (NCX) [1]. In teleosts, the gills (or the yolk skin in embryonic stages) are the main organs (over 90% of whole body) for Ca^2+^ uptake [3,15,27,28]. Epithelial ionocytes in the gills and the yolk skin are the specific cells for ion uptake [3,5,15]. In zebrafish, ionocytes originate from epidermal stem cells and Foxi3a and Foxi3b are the essential factors for ionocytes differentiation [29]. Zebrafish have at least five types of ionocytes; H^+^-ATPase-rich cells (HRCs), Na^+^-K^+^-ATPase-rich cells (NaRCs), Na^+^-Cl^−^-cotransporter-expressing cells (NCCCs), K^+^-secreting cells (KSCs), and SLC26-expressing cells (SLC26Cs) [16,30]. The different types of ionocytes have their own specific roles in ionregulation [15,16,31]. One of the ionocyte types, NaRCs, are specifically stained by the specific Na^+^-K^+^-ATPase (NKA) α-subunit antibody and are responsible for the Ca^2+^ regulation (Figure 1) [5,15].

Unlike mammals, zebrafish and other teleosts have only one TRPV5/6 ortholog, epithelium Ca^2+^ channel (ECaC) [5,8,15]. Furthermore, co-localization of zebrafish *ecac* and NKA was demonstrated by using in situ hybridization (ISH) and immunocytochemistry (ICC) [8]. This indicated the specific expression of ECaC in NaRCs. Other transepithelial Ca^2+^ transporters, the plasma membrane Ca^2+^-ATPase (PMCA) and Na^+^/Ca^2+^ exchanger (NCX), were also identified in zebrafish NaRCs [10]. There are six isoforms of PMCA and seven isoforms of NCX identified in zebrafish, and only NCX1b and PMCA2 are co-expressed with ECaC in the same ionocytes [10]. Among six distinct NKA α-subunit genes in zebrafish, only atp1a1a.5 is expressed in ECaC-expressing ionocytes [11,32]. Liao et al. revealed that atp1a1a.1 expression in zebrafish was stimulated by acclimation to a low Ca^2+^ water, suggesting that atp1a1a.1 provides the Na^+^ gradient to drive the operation of NCX1b [11]. Taking these results together, the model of transepithelial Ca^2+^ transport in zebrafish NaRC is similar to that in the kidneys and intestine of mammals.

The ability of Ca^2+^ uptake is elevated following development of zebrafish [8]. At the same time, ECaC mRNA expression is gradually increased [8]. When zebrafish embryos were treated with low- (0.02 mM) and high-Ca^2+^ water (2 mM Ca^2+^), respectively, the low-Ca^2+^ treatment stimulated the density of *ecac*-expressing cells, ECaC mRNA expression, and Ca^2+^ uptake [8,18,33,34]. In adult zebrafish gills, ECaC mRNA expression was also stimulated by low Ca^2+^ water treatment [10,34]. Additionally, in vitro study revealed that zebrafish ECaC is a calcium-selective channel capable of inward calcium transport at physiological Ca^2+^ concentrations [35], and ECaC knockdown resulted in decreased whole-body Ca^2+^ content in zebrafish embryos [18]. However, the mRNA expression of zebrafish PMCA2 and NCX1b in the gills and embryonic skin appears not to be regulated by the surrounding Ca^2+^ levels [10,18,33,34]. It was proposed that PMCA2 and NCX1b, having a constant-state expression under different ambient Ca^2+^ levels, play a supporting role for transepithelial Ca^2+^ absorption [15]. Plasma Ca^2+^ level is maintained around a value of 2.5 mM in vertebrates, but zebrafish continually face changes in environmental Ca^2+^ concentrations (FW [Ca^2+^], ~0.01–3 mM) [1,8]. In addition, intracellular Ca^2+^ levels are far lower than extracellular Ca^2+^ levels; the intracellular Ca^2+^ level of gill cells in fish is presumably less than 1 µM [1,3]. For the maintenance of Ca^2+^ homeostasis, it is vital to regulate Ca^2+^ uptake through modulating the ECaC in NaRC of zebrafish.

Examining the transepithelial Ca^2+^ transport in mammals is challenging because TRPV5 and TRPV6, the major transporters for Ca^2+^ reabsorption in the kidney, preferentially form the heteromultimers that exhibit functional redundancy and compensation [36,37,38]. Zebrafish possess only a single ortholog of mammalian TRPV5/6 and may be a more appropriate choice to explore the related issue. Therefore, some studies explored the effect of transpithelial Ca^2+^ transport on bone formation, development, and Ca^2+^ homeostasis by using ECaC-mutant lines or knocking down the ECaC expression in zebrafish [18,35]. In mammals, the expression of NCX1 is universal and especially dominant in vital organs such as the heart [39]. In zebrafish, there are two paralogs of mammalian NCX1, NCX1a and NCX1b. NCX1a is a heart-specific isoform and related to syndrome of cardiac fibrillations in zebrafish [40]. In contrast to NCX1a, NCX1b is not expressed in zebrafish heart [10,40]. Zebrafish NCX1b is expressed in NaRCs and is related to the Ca^2+^ extrusion in NaRCs [10]. Therefore, the subtle difference of zebrafish Ca^2+^ transporters from that of mammals may allow the use of the zebrafish model to explore the role of NCX in transepithelial Ca^2+^ transport without potential lethal problems.

### 2.2. Ca^2+^ Excretion in Zebrafish

In mammals, regulation of the property and/or expression of tight junction and TRPV5 in specific segments of the renal tubule are related to the Ca^2+^ excretion [1]. Although gene expression of many claudin proteins was identified in ion regulatory organs such the gills, kidneys, and skin in zebrafish, their function in Ca^2+^ permeability is still unclear [7]. On the other hand, ECaC expression was also detected in the kidneys in zebrafish [8]. Previous studies indicated the mRNA expression of ECaC in zebrafish embryos was differently regulated by external Ca^2+^ levels [8,18,19,21,33,34]. The renal ECaC expression may be modulated in zebrafish upon experiencing a Ca^2+^ challenge, which then further affects Ca^2+^ reabsorption. Thus, it results in changes to Ca^2+^ excretion. Nevertheless, this issue still requires evidence to support this hypothesis.

## 3. The Regulation of Ca^2+^ Uptake in Zebrafish

For body fluid Ca^2+^ homeostasis, Ca^2+^ absorption and excretion in Ca^2+^-regulation organs are modulated by a complex hormonal network in vertebrates. The action of calciotropic hormones and their receptors has been elucidated one after another in zebrafish in the past ten years [15,16]. In this section, we will first emphasize the actions of cortisol, vitamin D, stanniocalcin (STC-1), calcitonin (CT), parathyroid hormone (PTH), isotocin, hydrogen sulfide (H_2_S), and insulin-like growth factor 1 (IGF-1) on the regulation of Ca^2+^ uptake in zebrafish and the regulatory mechanism behind these actions. Finally, we will introduce the role of calcium-sensing receptor (CaSR) in the regulation of calciotropic hormones, and the mutual interplay of those hormones in terms of body fluid Ca^2+^ homeostasis in zebrafish.

### 3.1. The Hormonal Control of Ca^2+^ Uptake

#### 3.1.1. Cortisol

In mammals, corticoidsteroid hormones, including mineralcorticoid (aldosterone) and glucocorticoid (cortisol), are produced in the adrenal cortex of mammals and mineralcorticoid is the major player responsible for body fluid ion regulation [41,42]. Teleosts are unable to synthesis the aldosterone because of the lack of aldosterone synthase [43]. Therefore, cortisol functions as the aldosterone in teleosts [3]. In teleosts, corticoid steroid hormones are mainly synthesized in interrenal tissue of the head kidneys [44]. According to studies of cell experiments, cortisol is able to stimulate the transcription activity of reporter construct with the teleost glucocorticoid receptor (GR) and mineralcorticoid receptor (MR) [45,46,47,48]. Nevertheless, in vivo study indicated that cortisol regulates body fluid ion homeostasis via GR rather than MR in zebrafish [33,49,50,51,52].

Treatment of cortisol analogs decreased Ca^2+^ absorption in mammals through suppressing the expression of TRPV6 and calbindin-D9K in the kidney and duodenum [53,54]. However, cortisol was shown to be a hypercalcemic hormone in fishes [13,33,55,56]. In zebrafish, low Ca^2+^ water treatment increased the mRNA expression of 11β-hydroxylase, the final-step enzyme for cortisol synthesis, and exogenous cortisol treatment stimulated Ca^2+^ uptake in zebrafish embryos [33]. Moreover, cortisol treatment stimulated the mRNA expression of ECaC, but not that of PMCA2 and NCX1b, in zebrafish [33]. In the zebrafish morphants with GR translational knockdown, cortisol treatment did not stimulate ECaC transcript and Ca^2+^ uptake. On the contrary, MR knockdown did not affect the hypercalcemic action of cortisol on zebrafish [33]. Additionally, the localization of GR in NaRCs was demonstrated by using the ISH and/or ICC, and putative glucocorticoid-responsive element (GRE) was identified in the promoter region of ECaC in zebrafish [33,50]. Hence, cortisol may stimulate Ca^2+^ uptake through directly upregulating ECaC mRNA expression. On the other hand, cortisol was also found to positively regulate gene expression of the receptor and the synthesis enzyme of vitamin D [33]. As such, cortisol may be able to indirectly regulate Ca^2+^ uptake through stimulating other hypercalcemic hormones.

In zebrafish, ionocytes are derived from epidermal stem cells and Foxi3a and Foxi3b are the essential transcription factors for ionocytes differentiation [29]. Previous studies indicated that cortisol treatment increased the density of NaRCs but did not change the density of epidermal stem cells, and GR knockdown resulted in the decreased density of NaRCs in zebrafish [50,57]. Furthermore, the mRNA expression of Foxi3a and Foxi3b was upregulated in both zebrafish embryos and the cultured gills after cortisol treatment [50,57]. Therefore, cortisol-GR signaling is involved in the stimulation of Ca^2+^ uptake through inducing ECaC mRNA expression and NaRCs differentiation/differentiation.

#### 3.1.2. Vitamin D

There are two vital enzymes, vitamin D-25 hydroxylase (CYP2R1) and 1α-OHase (CYP27B1), for the synthesis of vitamin D in mammals. CYP2R1 in the liver converts vitamin-D precursor into 25-hydroxyvitamin D_3_ (25(OH)D_3_), which is then converted to 1α,25-dihydroxytamin D_3_ (1α,25(OH)_2_D_3_), the biological active form of vitamin D, by CYP27B1[58]. In contrast to CYP2R1 and CYP27B1, 25-hydroxyvitamin D_3_-24-hydroxylase (CYP24A1) degrades 1α,25(OH)_2_D_3_ in the peripheral tissues to maintain the level of vitamin D [58,59]. On the other hand, exogenous 1α,25(OH)_2_D_3_ treatment caused a feedback regulation for the maintenance of 1α,25(OH)_2_D_3_ level homeostasis through suppressing CYP27B1 and increasing CYP24A1 mRNA expression, respectively, in mammalian experiments [60,61,62,63]. In fishes, the genes of CYP24A1, CYP2R1, and CYP27B1 have also been identified, and 25(OH)D_3_ and 1α,25(OH)_2_D_3_ were measured in the plasma [64,65,66]. In a zebrafish study, exogenous 1α,25(OH)_2_D_3_ treatment was also found to decrease CYP27B1 and increase CYP24A1 mRNA expressions, respectively [34]. Hence, the feedback regulation of vitamin D synthesis appears to be conserved from zebrafish to mammals.

**Figure 2:**
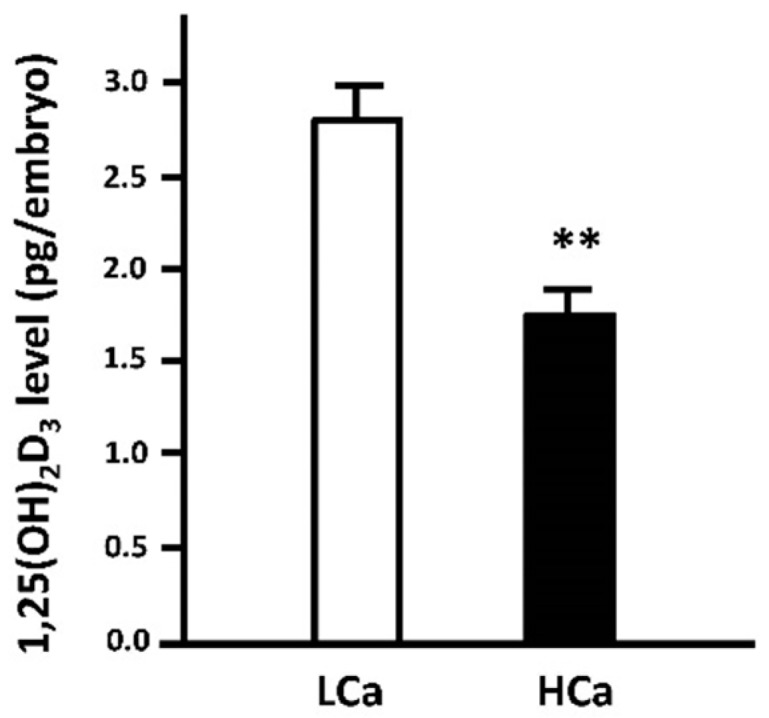
Effect of environmental Ca^2+^ concentrations on whole-body 1,25-dihydroxyvitamin D_3_ (1,25(OH)_2_D_3_) content in 3 day post-fertilization (dpf) zebrafish embryos. One to two cell-stage zebrafish embryos were incubated in low- (0.02 mM Ca^2+^) and high-Ca^2+^ (2.0 mM Ca^2+^) water, respectively, for three days. The low-Ca^2+^ (LCa) group showed a higher level of 1,25(OH)_2_D_3_ than the high-Ca^2+^ (HCa) group. Whole-body 1,25(OH)_2_D_3_ content was measured with an ELISA kit (E0467GE, EIAlab, Wuhan, China). Values are the mean ± SEM (*n* = 5). Student’s *t*-test, ** *p* < 0.01.

Lin et al. reported stimulation of the mRNA expression of both CYP2R1 and vitamin D receptor (VDR) by low-Ca^2+^ water treatment in zebrafish [34]. Low-Ca^2+^ water treatment stimulated whole-body 1α,25(OH)_2_D_3_ concentration but did not affect CYP27B1 mRNA expression in zebrafish (Figure 2) [34]. Incubation with 1α,25(OH)_2_D_3_ was found to increase whole-body calcium content and Ca^2+^ uptake in zebrafish through upregulation of ECaC (but not PMCA2 and NCX1b) mRNA [34]. Hence, vitamin D functions as a hypercalcemic hormone in zebrafish. Vitamin D modulates many physiological functions via VDR. There are two paralogous VDRs, VDRa and VDRb, in zebrafish [34]. Knockdown of VDRa, but not that of VDRb, resulted in the downregulation of Ca^2+^ uptake and ECaC mRNA expression in zebrafish [34]. Additionally, localization of VDRa and NaRCs reinforced the actions of vitamin D on zebrafish body fluid Ca^2+^ homeostasis [34]. Taken all together, vitamin D-VDRa signaling may directly induce ECaC mRNA expression to upregulate the capacity of Ca^2+^ uptake.

#### 3.1.3. Parathyroid Hormone (PTH)

**Figure 3:**
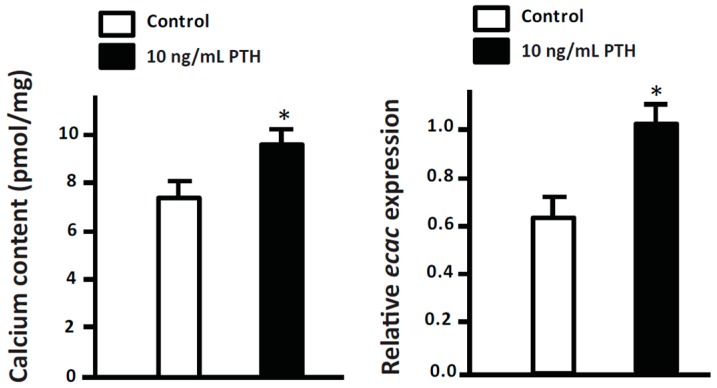
Effect of human parathyroid hormone (PTH) amide on whole-body ECaC mRNA expression and calcium content in 3-dpf zebrafish embryos. One to two cell-stage zebrafish embryos were treated with 10 ng/mL human PTH 1-34 amide (H-5964, BACHEM, Torrance, CA, USA) for three days. The treatment of human PTH 1-34 amide resulted in significant increases in calcium content and ECaC mRNA expression. Whole-body Ca^2+^ content was measured by atomic absorption spectrophotometry. mRNA expression was analyzed by qPCR using the primer sets in the study by previous study [21], and the values were normalized to β-actin expression. Values are the mean ± SEM (*n* = 5). Student’s *t*-test, * *p* < 0.05.

PTH is expressed and secreted from the parathyroid gland (PTG) in mammals [1]. The PTG is absent in fish; however, the expression of PTH was detected by RT-PCR, ICC, and/or ISH in several tissues of zebrafish, such as the gills, muscle, neuromast of lateral line, and brain [21,67,68]. There are two paralogs of PTH, PTH1 and PTH2, in zebrafish [67]. Diluted FW with additional supplement of CaCl_2_ was reported to inhibit PTH1 mRNA expression in zebrafish embryos [69]. On the contrary, PTH1 mRNA expression in zebrafish embryos was stimulated by low ambient Ca^2+^ level [21,26,68]. PTH1 positively regulates Ca^2+^ absorption and ECaC mRNA expression in zebrafish embryos based on PTH1 gain- and loss-of-function experiments [21]. In addition, treatment with human PTH (1-34 amide) was shown to increase Ca^2+^ absorption and ECaC mRNA expression in zebrafish (Figure 3). In a recent study, knockdown of PTH1 in zebrafish significantly downregulated the number of epidermal stem cells and caused a concomitant decrease in the number of NaRCs [68]. Therefore, PTH1 enhances Ca^2+^ absorption by stimulating ECaC mRNA expression and the proliferation/differentiation of NaRCs in zebrafish. In contrast to PTH1, PTH2 is not modulated at the translation level by ambient Ca^2+^ level [21,68]. Supporting the above finding, both the calcium content and the gene expression of Ca^2+^ transporters (ECaC, PMCA2, and NCX1b) in zebrafish were not changed by PTH2 gain- or loss-of-function [21]. Therefore, only PTH1 is involved in the regulation of the Ca^2+^ uptake mechanism in zebrafish.

There are three types of PTH receptors, PTH1R, PTH2R, and PTH3R, in zebrafish, and zebrafish PTH1 was capable of activating the PTH1R and PTH3R in vitro study [70]. PTH1R and PTH3R are expressed in zebrafish gills, but the existence of PTH1R and/or PTH3R in NaRCs is still unknown [68]. In addition, there is no physiological study to clarify the role of these PTH receptors in body fluid Ca^2+^ homeostasis, and this issue remains to be explored in the future.

#### 3.1.4. Stanniocalcin-1 (STC-1)

STC-1 is a homodimeric glycoprotein hormone and was first identified in fish [71]. In mammals, STC functions as a paracrine and autocrine and has a universal expression in a range of tissues [71]. Heterogeneous expression of human STC-1 in rat inhibited the intestinal calcium absorption, but the regulatory mechanism was not clear [72]. In fish, STC-1 is expressed in the corpuscle of Stannius (CS), a fish-specific endocrine gland attaching to the kidneys. Removing the CS from FW eel resulted in hypercalcaemia [73]. In contrast, intra-arterial injection of CS crude homogenate in trout caused an inhibitory effect on Ca^2+^ uptake [74].

STC-1 expression in zebrafish was significantly stimulated by high-Ca^2+^ water treatment, and knockdown of STC-1 enhanced zebrafish Ca^2+^ uptake [18]. ECaC, but not PMCA2 or NCX1b, appears to be the regulatory target of the actions of STC-1 in zebrafish according to the finding that only the expression of ECaC was decreased by STC-1 overexpression [18,24]. To reinforce this notion, STC-1 transcript was increased in ECaC zebrafish morphants and ECaC mutant zebrafish [18,35]. Thus, STC-1 is a hypocalcemic hormone to suppress Ca^2+^ uptake function by downregulating ECaC mRNA expression. STC-1 was recently demonstrated to decrease the capacity of Ca^2+^ uptake through negatively regulating the number of zebrafish NaRCs, which is probably mediated by the transcription factor of Foxi3a [24]. This provides a new insight into the molecular mechanism of negative action of STC-1 on the transepithelial Ca^2+^ uptake. STC receptor has not been identified in animals so far, and this is a limitation to further understand the action of STC-1 on Ca^2+^ regulation in mammals.

#### 3.1.5. Calcitonin

Calcitonin (CT) is a type of small peptide produced in parafollicular C cells of the thyroid gland and may function as a hypocalcemic hormone in mammals [75]. However, some studies have shown the hypercalcemic action of CT in mammals. Incubation of CT was able to increase Ca^2+^ absorption by stimulating NCX activity in the distal tubules of rabbit nephron [76]. In human, infusion of particular CT concentrations induced hypercalcemia and increased renal Ca^2+^ reabsorption [77]. As such, the hypocalcemic function of CT is somewhat controversial in mammals [74]. In fish, CT is mainly synthesized in the ultimobranchial gland (UBG), the small body of the pharynx [3]. Hypocalcemic action of CT was reported in several fish species [78,79,80].

In zebrafish, the mRNA expression of CT in embryos and adult UBG was significantly stimulated by high-Ca^2+^ water treatment. The treatment of high-Ca^2+^ water also increased the mRNA expression of CT receptor (CTR) in embryos and adult gills [19]. Overexpression of CT resulted in a decrease of ECaC and an increase of STC-1, respectively, at the mRNA level in zebrafish embryos [19]. Therefore, CT regulates Ca^2+^ absorption probably through directly or indirectly (via STC-1) downregulating ECaC expression. Notably, overexpression of CT induced a short-term hypocalcemia initially and a long-term hypercalcemia subsequently in zebrafish embryos [19]. The increased mRNA expression of PMCA2 and NCX1b, probably a result of the upregulated expression of VDR and PTH receptor, was suggested to contribute to long-term hypercalcemia [19]. These findings offer a new insight into the biphasic effects of CT on body fluid Ca^2+^ homeostasis in vertebrates.

#### 3.1.6. Isotocin

Isotocin is the teleost homologue of mammalian oxytocin gene [81,82]. Oxytocin is involved in many physiological functions, and is primarily produced in neurons of the hypothalamo-neurohypophysial system in mammals [83]. In fishes, isotocin is expressed in the brain and others tissues including the gills, ovary, muscle, and skin [20,84,85,86,87]. In zebrafish, mRNA expression of isotocin was dominantly upregulated after acclimation to ion-poor water, and loss-of-function of isotocin resulted in a decrease of whole-body calcium content with concomitant declines in ECaC mRNA expression and the number of NaRCs [20]. The mechanism behind this regulation is associated with the decrease in the number of epidermal stem cells and Foxi3a mRNA expression [20]. Therefore, isotocin appears to positively stimulate Ca^2+^ uptake function through increasing ionocytes differentiation and proliferation. In zebrafish, isotocin receptors were identified and the expression was detected in gills [20]. Mammalian oxytocin could modulate endocrine action on osmoregulation in mammals [83]. The discovery of zebrafish isotocin’s action on Ca^2+^ uptake provides some clues to explore novel functions of mammalian oxytocin in terms of body fluid ionic homeostasis.

#### 3.1.7. Hydrogen Sulfide (H_2_S)

H_2_S is a gaseous transmitter and there are two vital cytosolic enzymes, cystathionine-γ-lyase (CSE) and cystathionine-β-synthase (CBS), for the production of endogenous H_2_S [88,89,90]. These two enzymes are universally expressed in various tissues in mammals. H_2_S is involved in many physiological activities including cardiovascular regulation, cytoprotection, inflammation, and cell energy production [90]. Additionally, several studies revealed that treatment with H_2_S donors increased the intracellular Ca^2+^ level in rat microglial cells, rat aorta endothelial cells, and HeLa cells [91,92,93]. In zebrafish embryos incubated with low-Ca^2+^ water, the treatment of H_2_S donors increased whole-body Ca^2+^ uptake and/or calcium content [23], suggesting the action of H_2_S on body fluid Ca^2+^ homeostasis. Both CBSb and CSE are expressed in zebrafish NaRCs, but only CBSb mRNA expression is stimulated by low Ca^2+^ water treatment [23]. Knockdown of CBSb, but not CSE, evidently decreased the Ca^2+^ absorption of zebrafish in low Ca^2+^ water. Supporting this loss-of-function result, only the pharmacological inhibition of CBS activity resulted in decreased Ca^2+^ uptake in zebrafish [23]. There are several putative PKA phosphorylation sites in mammalian TRPV5 and activation of these sites can enhance channel activity [94,95]. Likewise, H_2_S, generated by CBSb in NaRCs, may activate ECaC activity through the PKA-cAMP pathway and then upregulate Ca^2+^ absorption in zebrafish based on the pharmacological experiments with the relevant inhibitors.

#### 3.1.8. Insulin-Like Growth Factor 1 (IGF-1)

In mammals, IGF-1, secretion from the liver and/or the local cells, regulates Ca^2+^ absorption in the kidneys through increasing vitamin D synthesis, and the physiological actions of IGF-1 are mediated by the IGF-1 receptor (IGF-1R) [1,96,97]. Mammalian IGF-1R is expressed throughout the nephron, but the direct effect of IGF-1 on Ca^2+^ uptake requires further clarification [98,99,100,101,102]. In fish, IGF-1 expression was identified in the branchial ionocytes of tilapia [103,104]. Injection of IGF resulted in upregulation of both ionocytes’ development and Na,K-ATPase expression in brown trout [105]. IGF-1 is able to stimulate zebrafish cell proliferation through activating the PI3K-Akt signaling pathway [106]. In zebrafish embryos, a phospho-Akt signal was detected in NaRC by ICC [22], and lower-Ca^2+^ water treatment increased the number of phospho-Akt signals and NaRCs [22]. Subsequent pharmacological experiments suggested that the IGF-1R-PI3K-Akt signaling pathway directly regulates Ca^2+^ absorption through stimulating NaRC proliferation [22]. These findings bring a novel insight into the action of IGF-1 signaling on body fluid Ca^2+^ homeostasis in vertebrates.

### 3.2. Calcium-Sensing Receptor (CaSR)

**Figure 4:**
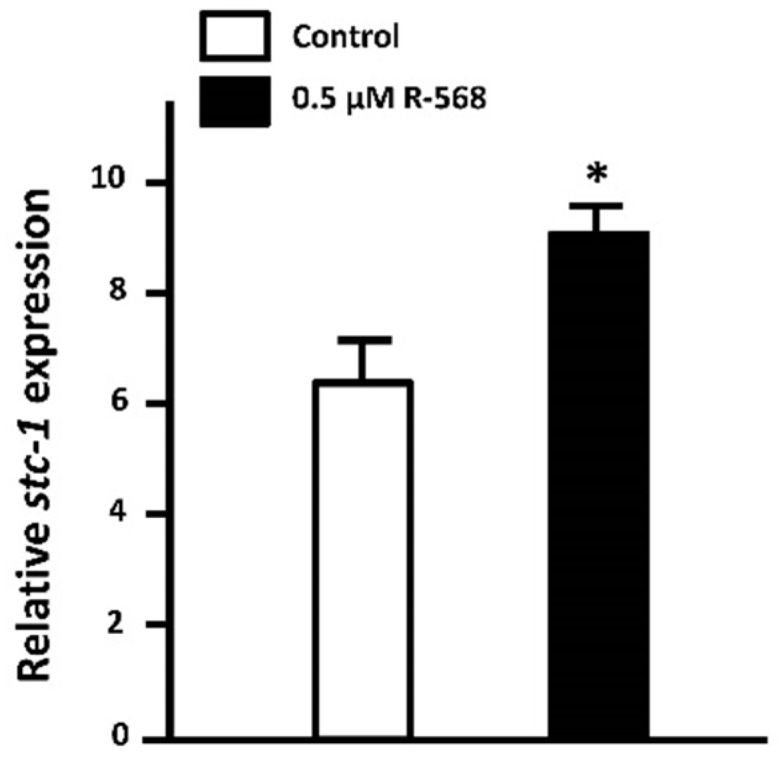
Effect of activated calcium-sensing receptor (CaSR) on STC-1 mRNA expression in 3-dpf zebrafish embryos. Zebrafish embryos at 3-dpf were treated with 0.5 µM R-568 (sc-361302, Santa Cruz Biotechnology, Santa Cruz, CA, USA) for 8 h. The treatment of R-568 significantly stimulated STC-1 mRNA expression. mRNA expression was analyzed by qPCR using the primer sets in the study by previous study [21], and the values were normalized to β-actin expression. Values are the mean ± SEM (*n* = 5). Student’s *t*-test, * *p* < 0.05.

The expression and/or secretion of calciotropic hormones are dependent on extracellular Ca^2+^ level. CaSR, a G protein-coupled receptor, senses the change in extracellular Ca^2+^ level and is activated by increased Ca^2+^ levels in mammals. Activated CaSR differently modulates the calciotropic hormones (i.e., PTH, CT and vitamin D) for the maintenance of body fluid Ca^2+^ homeostasis [25]. In fish, CaSR also functions as a Ca^2+^ sensor and is activated by the increased extracellular Ca^2+^ level. In in vitro study, the activation of tilapia CaSR was enhanced within 5 min when the extracellular Ca^2+^ level was raised from 0.5 to 2 or 3 mM [107,108]. Additionally, the expression of fish CaSR was identified in several hormone-secreting organs [21,26,109,110]. In zebrafish, knockdown of CaSR caused defects in Ca^2+^ absorption, skeletal development, and the gene expression of calciotropic hormones and ECaC [21,26,111]. Furthermore, activated CaSR exerts different actions on PTH1, STC-1, and CT in zebrafish [21,26]. CaSR is expressed in PTH1-expressing tissues (i.e., the gills and the neuromast of lateral line) and the CS in zebrafish [21,26]. CaSR knockdown resulted in increased PTH1 mRNA expression in zebrafish under either a low or high Ca^2+^ water situation [21,26], indicating the negative action of activated CaSR on PTH1. In contrast to PTH1 expression, STC-1 mRNA expression was short-term downregulated in zebrafish *casr* morphants [21]. Pharmacological experiments with R568, an allosteric agonist of CaSR, caused upregulation of STC-1 mRNA expression in zebrafish embryos (Figure 4), demonstrating the positive action of activated CaSR on STC-1 in zebrafish. On the other hand, the treatment of CaSR agonist did not stimulate CT secretion in rainbow trout [109]. In zebrafish, GCM2 gain- and loss-of function resulted in upregulation and downregulation, respectively, of CaSR mRNA expression, which was upregulated and downregulated, respectively, although the CT mRNA expression was not changed [112]. CT mRNA expression was not regulated by knockdown experiment of CaSR in zebrafish [21].Taken all together, activated CaSR appears not to be involved in the regulation of CT in zebrafish.

**Figure 5:**
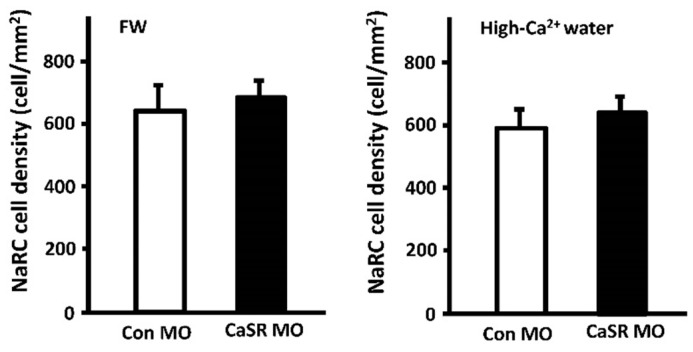
Effect of CaSR knockdown on the density of NaRCs in 3-dpf zebrafish embryos. CaSR and control morpholinos (CaSR and Con MO) [21], respectively, were microinjected into 1–2 cell-stage embryos incubated in normal freshwater (FW) or high-Ca^2+^ water (2.0 mM Ca^2+^). CaSR knockdown did not regulate the density of NaRCs. Cell counting for the density of NaRCs followed the method of previous study [24]. Values are the mean ± SEM. (*n* = 12).

Several studies have revealed the role of CaSR in regulating cell proliferation in mammals [113,114,115]. In zebrafish, CaSR expression was detected in NaRCs by ICC, but knockdown of CaSR did not change the number of NaRCs in zebrafish embryos (Figure 5) [26]. CaSR knockdown in zebrafish was known to eventually stimulate the mRNA expression of both PTH1 and STC-1, which show reverse influence on NaRCs differentiation [21,24,26,68]. Taking all of this into account, no effect of CaSR knockdown on the number of NaRCs may reflect a counterbalance between the actions of PTH1 and STC-1. On the other hand, CaSR is co-expressed with TRPV5 in DCT/CNT, the specific segments for Ca^2+^ reabsorption, in the kidney, and activated CaSR is able to directly regulate TRPV5 activity in mammals [116]. The effect of activated CaSR on zebrafish ECaC activity is still unknown, and is an important issue to be studied in the future.

### 3.3. Mutual Counterbalance of Calciotropic Hormones

Body fluid Ca^2+^ homeostasis is tightly regulated by the crosstalk of calciotropic hormones in mammals. In zebrafish, the interplay between calcitropic hormones is a rising topic which has been recently discussed in a few studies. In a study by Lin et al., knockdown of CaSR caused a short-term decrease and a subsequent recovery/upregulation in STC-1 mRNA expression in zebrafish embryos; however, the PTH1 mRNA expression was continuously stimulated in zebrafish *casr* morphants [21]. As PTH1 expression was downregulated in zebrafish *casr* morphants, the long-term expression of STC-1 mRNA was no longer upregulated. Furthermore, PTH1 gain-of-function in zebrafish stimulated STC-1 mRNA expression [21]. Therefore, upregulated STC-1 expression in zebrafish *casr* morphants may be eventually abolished by the increased PTH1. Reinforcing this notion, STC-1 gain-of-function in zebrafish enhanced PTH1 mRNA expression [21]. Based on these results, there is a mutual counterbalance between the expression, release, and/or action of PTH1 and STC-1, and this interplay is important for the maintenance of body fluid Ca^2+^ homeostasis [21].

Cortisol via GR positively regulates the gene expression of the receptor and the synthesis-enzyme of vitamin D in zebrafish [33]. Exogenous cortisol treatment inhibited and increased STC-1 and CT mRNA expressions, respectively, in zebrafish embryos [112]; however, a previous study indicated that increased CT expression was capable of stimulating STC-1 mRNA expression in zebrafish [19]. The finding of upregulated CT expression and decreased STC-1 expression in zebrafish embryos with cortisol treatment implies a subtle interplay among cortisol, vitamin D, STC-1, and CT, which awaits further studies in the future.

## 4. Conclusions and Perspectives

**Figure 6:**
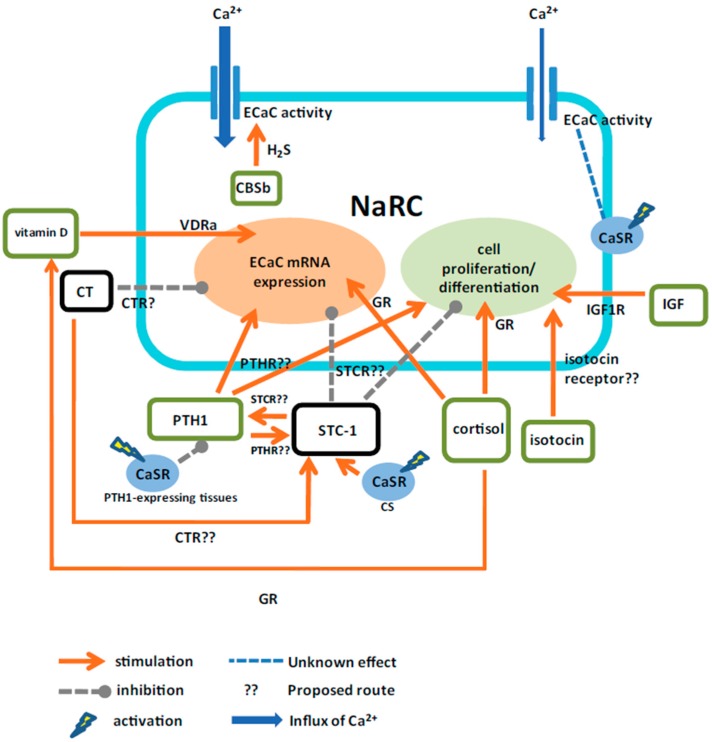
A proposed model for the actions of calcitropic hormones on zebrafish NaRCs. Calciotropic hormones are suggested to directly and indirectly (via the effector hormone(s)) regulate the cell proliferation/differentiation and the mRNA expression or activity of ECaC in NaRCs. CaSR, calcium-sensing receptor; CBSb, cystathionine-β-synthaseb; CS, corpuscle of Stannius; CT, calcitonin; CTR, calcitonin receptor; ECaC, epithelial Ca^2+^ channel; GR, glucocorticoid receptor; H_2_S, hydrogen sulfide; IGF, insulin-like growth factor; IGF1R, insulin-like growth factor 1 receptor; NaRC, Na^+^-K^+^-ATPase-rich cell; PTH1, parathyroid hormone 1; PTHR, parathyroid hormone receptor; STC-1, stanniocalcin1; STCR, stanniocalcin receptor; VDRa, vitamin D receptor A.

The active transepithelial Ca^2+^ transport, being carried out through apical ECaC or TRPV5/6 and basolateral PMCA and NCX, is conserved between zebrafish NaRCs and the specific cells of mammalian kidney and intestine. As discussed in the present review, zebrafish has been an emerging animal model to explore the mechanisms associated with Ca^2+^ uptake and hormonal control, and the findings in zebrafish provide new insight into the related issues in vertebrates. Hormones via their receptor(s) regulate Ca^2+^ uptake through direct (via regulating Ca^2+^ transporter) and/or indirect (via modulating the other calciotropic hormones) routes (Figure 6). However, the evidence for the receptors of some hormones in NaRCs in zebrafish are still unclear. Additionally, more research is needed to elucidate the mutual counterbalance among calciotropic hormones (and the receptors) in zebrafish.
